# Design, Implementation and Maintenance of a Model Organism Database for *Arabidopsis thaliana*

**DOI:** 10.1002/cfg.408

**Published:** 2004-06

**Authors:** Danforth Weems, Neil Miller, Margarita Garcia-Hernandez, Eva Huala, Seung Y. Rhee

**Affiliations:** 1 National Center for Genome Resources 2935 Rodeo Park Drive East Santa Fe NM 87505 USA; 2 The Arabidopsis Information Resource Carnegie Institution of Washington Department of Plant Biology 260 Panama Street Stanford CA 94305 USA

## Abstract

The *Arabidopsis* Information Resource (TAIR) is a web-based community database for the model plant *Arabidopsis thaliana*. It provides an integrated view of genes, sequences, proteins, germplasms, clones, metabolic pathways, gene expression, ecotypes, polymorphisms, publications, maps and community information. TAIR is developed and maintained by collaboration between software developers and biologists. Biologists provide specification and use cases for the system, acquire, analyse and curate data, interact with users and test the software. Software developers design, implement and test the database and software. In this review, we briefly describe how TAIR was built and is being maintained.


**Comparative and Functional Genomics**
*Comp Funct Genom* 2004; **5**: 362–369.
Published online in Wiley InterScience (www.interscience.wiley.com). **DOI:** 10.1002/cfg.408

**Review**

### Design, implementation and maintenance of a model organism database for *Arabidopsis thaliana*

Danforth Weems[1], Neil Miller[1], Margarita Garcia-Hernandez[2], Eva Huala[2] and Seung Y. Rhee[2]*
[1] *National Center for Genome Resources, 2935 Rodeo Park Drive East, Santa Fe, NM 87505, USA*
[2] *Carnegie Institution of Washington, Department of Plant Biology, 260 Panama Street, Stanford, CA 94305, USA*

**Correspondence to: Seung Y. Rhee, The Arabidopsis Information Resource, Carnegie Institution of Washington, Department of Plant Biology, 260 Panama Street, Stanford, CA 94305, USA. E-mail: rhee@acoma.stanford.edu*

Received: 12 March 2004
Accepted: 12 March 2004

### Abstract

**The *Arabidopsis* Information Resource (TAIR) is a web-based community database for the model plant *Arabidopsis thaliana*. It provides an integrated view of genes, sequences, proteins, germplasms, clones, metabolic pathways, gene expression, ecotypes, polymorphisms, publications, maps and community information. TAIR is developed and maintained by collaboration between software developers and biologists. Biologists provide specification and use cases for the system, acquire, analyse and curate data, interact with users and test the software. Software developers design, implement and test the database and software. In this review, we briefly describe how TAIR was built and is being maintained. Copyright © 2004 John Wiley & Sons, Ltd.**

**Keywords:** **model organism database; *Arabidopsis thaliana*; bioinformatics; biological databases; web application; curation; user interface design**

### Introduction

Over 10 000 biologists around the world have adopted *Arabidopsis* as a model for studying many aspects of plant biology [7,10]. In addition, recent technologies enabling high-throughput analyses of the *Arabidopsis* genome, transcriptome and proteome are generating an unprecedented amount of information. It is expected that by the end of the year 2010, we will know the biochemical function, expression pattern, subcellular location, mutant and overexpression phenotypes, and interaction partners of nearly every *Arabidopsis* gene [12].

The completion of the *Arabidopsis* genome sequence has been followed by an effort to fully sequence the rice genome [4,15] and efforts are under way to sequence several other plant species [8,14]. In addition, large numbers of expressed sequence tag (EST) sequences are available for many economically important plant species. These sequences will provide direct links between the biology of the fully sequenced plants and the EST-rich species, thereby elucidating the comparable biological processes in economically important plant species. A Web-enabled *Arabidopsis* information resource that can facilitate the analysis and interpretation of the *Arabidopsis* genome, transcriptome and proteome and make the necessary connections to other plant information resources is an essential tool for researchers to make maximal use of the recent surge of information.

We developed TAIR to serve as a comprehensive Web-based information resource for the model plant *Arabidopsis thaliana*. Our primary goal in this work was to develop a new information infrastructure containing all available genomic and genetic data and make it accessible to the public through a set of user-friendly search, browse and visualization tools. This review summarizes how we designed, implemented and are maintaining TAIR.

### Materials and methods

### Database design

The TAIR database was designed in three iterative phases: conceptual design, logical translation and

Copyright © 2004 John Wiley & Sons, Ltd.

physical implementation. Conceptual design was carried out by biologists and was represented in Unified Modelling Language using GDPro (version 5.0). In building the conceptual model, established or emerging community standards were consulted when appropriate. For example, we adapted Object Management Group's Life Science Research working group's (**www.omg.org/lsr/**) model for representing genome maps in designing our MapElement and Assignment domains. Likewise, we consulted the Microarray Gene Expression Data Society's representation of microarray data (**www.mged.org/**) in designing the gene expression domain. Logical and physical implementation followed the standard principles of relational database design with an emphasis on normalization. As such, the data was decomposed into numerous tables, representing discrete units of information, and relationships between the tables were created through the use of primary/foreign keys pairs. ERWin (version 4.0) was used to implement and maintain the database schema.

The schema is hierarchical–relational, where hierarchical relationships are utilized to allow for greater normalization of the data and minimization of linking tables. An example of this hierarchical–relational design is the TairObject hierarchy (Figure 1A). TairObject is a parent table that is an umbrella entity for many data objects in TAIR. A few of the child tables associated with TairObject include NucleotideSequence, Gene, Clone, GeneticMarker, and Assignment. All of these data types can have ancillary data associated with them, such as attributions about who provided the data, external links to other websites, associations with publications, etc. Since the primary key within TairObject is inherited as foreign keys in all child tables of TairObject, this key allows for a reduction in the number of linking tables. This approach has provided a logical design that facilitates understanding of the data relationships. Other examples of hierarchical relationships in TAIR include the Community, Reference and MapElement parent tables (Figure 1B). Community includes Person and Organization data. Reference associates Publications, DatabaseReferences, AnalysisReferences and Communications.

MapElement illustrates that a child table can itself be a parent table for other data types. MapElement represents those members of TairObject that can be mapped relative to each other. This

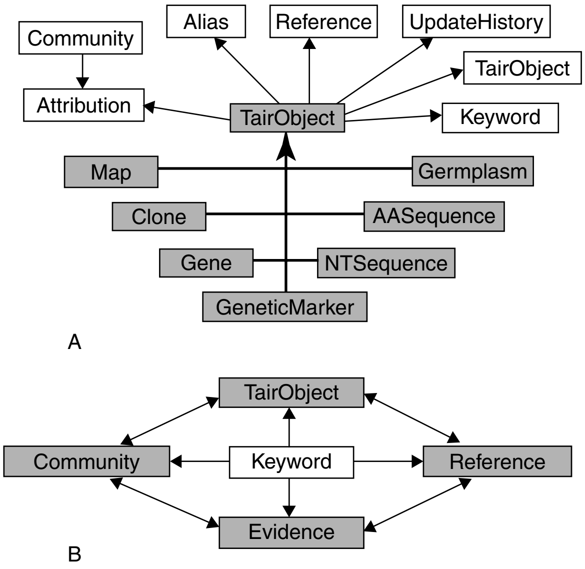

**Figure 1.** Key features of TAIR Database design concepts. (A) TairObject is a parental entity that inherits a number of data objects (grey boxes). This allows minimization of association tables (white boxes). This type of hierarchical–relational data structure is used throughout the database and allows flexible extension and high performance. (B) Highest level of data segregation in TAIR database. In addition to TairObject, we have Community, Reference and Evidence, which are the highest parental entities. They are associated with each other by several common association tables, the most significant one being keyword collection includes maps, contigs, clones, clone ends (e.g. BAC ends and ESTs), loci, genes, genetic markers and polymorphisms. All of these MapElement data types can be mapped onto one another via the Assignment table, which uses the MapElement primary key to define the mapping relationship.

The hierarchical–relational design approach implemented in TAIR is flexible enough to allow for growth as TAIR continues to include new data types. This design is also easily incorporated into the software process for providing access to the data.

### Software design

The TAIR website (**www.arabidopsis.org/**) is a gateway into the relational database which houses extensive information collected and curated by our staff. Since TAIR exists to serve the plant biology research community, it is essential that we make

Copyright © 2004 John Wiley & Sons, Ltd. 

the information stored in the database available to this community in the most useful and transparent way possible. To accomplish this, we developed a range of search and graphic display tools, search results pages and object detail pages designed with the perspective of the biologist in mind.

The design of TAIR's user interfaces has been primarily driven by the need to provide fast, easy access to all parts of our database for all researchers and students worldwide. This necessitates that the site maintains a high degree of cross-platform compatibility and performs well even when accessed using older browsers and operating systems. To satisfy these requirements we initially designed the TAIR pages using HTML (Hyper-Text Markup Language) for widest compatibility at the time the project began (1999). We also favoured light client and heavy server design and avoided stand-alone Java applications and applets to maximize accessibility of the site. We recently converted most TAIR pages from HTML to JSP (Java Server Pages), to more flexibly accommodate users of different backgrounds and interests by customizing pages to include only tools and data of interest.

In addition to this primary design consideration, other considerations have included maintaining a consistent look and feel and balancing complexity (availability of every possible feature and type of information) against simplicity and ease of use. To maximize the quantity of information presented without degrading the usability of the pages, we employed several strategies, including providing visual clues, selective display of datasets and details, and multiple views that emphasize different attributes of data. To further enhance usability for a variety of users, ranging from experienced *Arabidopsis* researchers to biologists working in other areas and students at various levels, we have included extensive help documentation and are currently developing a set of tutorials for the site.

### Implementation

TAIR system infrastructure has three components: database server, web server and analysis cluster (Figure 2A). TAIR database is implemented using Sybase version 12.5 and is currently running on Sun E450 ($4 \times 400$ Mhz processor, 2 GB RAM, 70 GB disk space). The web server uses Apache version 1.3.14. Analysis cluster is a parallel compute cluster consisting of multiple Intel machines running Linux (900 MHz processor, 1 GB RAM, 30 GB disk) and processes jobs for programs such as BLAST [3], PatMatch (**arabidopsis.org/cgi-bin/patmatch/nph-patmatch.pl**) and FASTA (**www.ebi.ac.uk/fasta33/**). Data exchange scripts are written in Perl (version 5.8.0) and the clustering programme is written in Python (version 2.2.2).

The TAIR software application allows users and curators to interact with the data in the database. It is created using Java Enterprise technologies such as servlets and Java Server Pages (JSPs). JRun is used as the servlet engine. Software can usually either be flexible or fast, since performance optimizations often increase the fragility of a system. Since the TAIR data model and application requirements are constantly evolving, the software design has consistently favoured flexibility over performance.

The TAIR software is implemented with the classic model/view/controller design (Figure 2B). The lowest level of the TAIR application is the TAIR Foundation Classes (Tfc) package. The Tfc package acts as a layer of abstraction (model) to the TAIR database by encapsulating the majority of database queries within its classes. At a higher level of abstraction immediately above the Tfc package are the 'querytools' classes (controller). These classes extend and contain the Tfc classes to form complex objects that are used to display detailed information on a given element and its associated data. The querytools classes are reused heavily throughout the application. The presentation layer is implemented using Java Server Pages (view) and is kept as simple as possible with a minimum of embedded code.

Quality assurance and testing are done on TAIR software in two main ways. Unit tests are performed on individual pieces of software, using a custom framework that allows testing of the basic functionality of each module of code. Large pieces of functionality, including new features, are tested through an iterative cycle using a test environment. Software updates are only pushed to production after they have gone through a review cycle through the test environment. Software updates are deployed using an inspection server, which allows for testing of the update in the production environment with minimal disruption of the production site.

Source code and binary files for TAIR software are available upon request. It is freely available

Copyright © 2004 John Wiley & Sons, Ltd. 

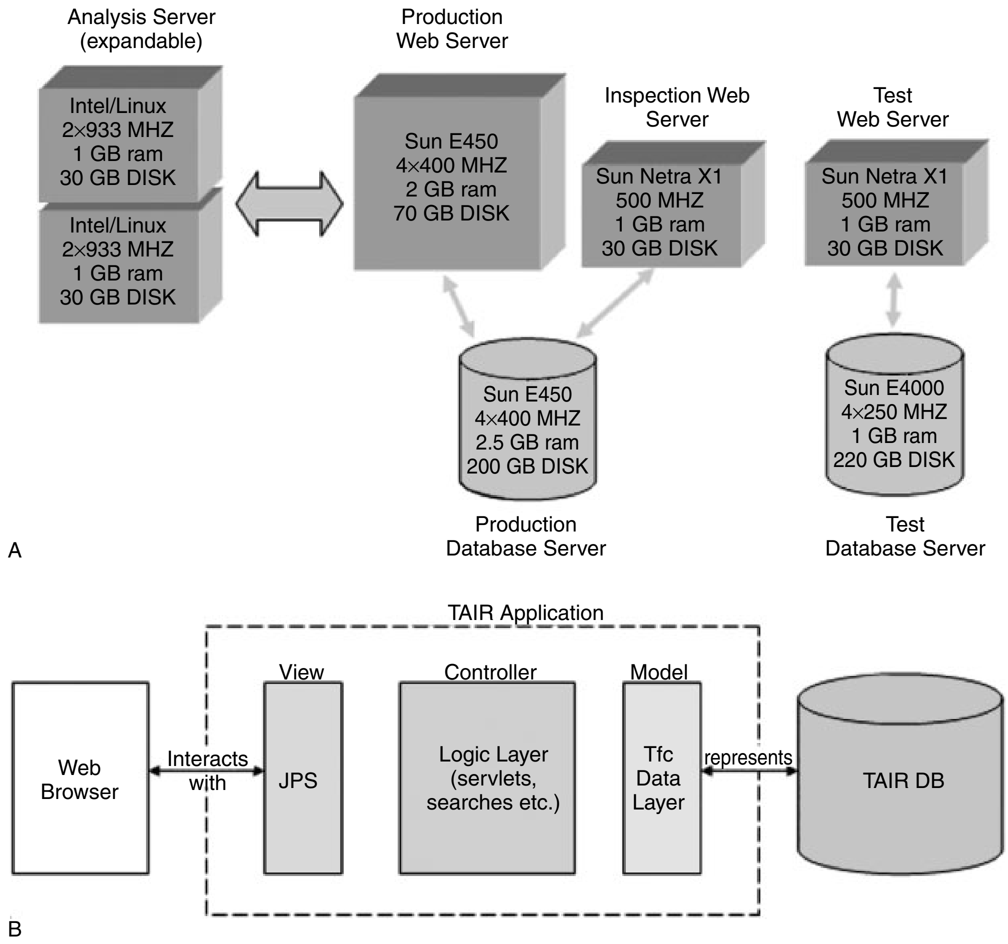

**Figure 2.** Hardware and software infrastructure. (A) Hardware set-up. (B) Software architecture. The Model/View/Controller design is implemented using a data layer, a logic layer and a Java Server Page (JSP) presentation layer. JSPs are kept as simple as possible with a minimum of embedded code for academic researchers. Source code licenses for TAIR software are available on-line (**arabidopsis.org/about/software.jsp**).

### Curation

Data curation is a crucial task in any data management system. Curation involves organizing the data into logical collections (data types), establishing relationships among data types, collecting metadata (descriptive information about the data), defining metadata standards, transforming data into standard formats, describing data in consistent and standard ways, and assessing data quality. All the data in TAIR are subjected to curation. We use a number of different strategies for curation, including computational, manual, in-house and external (community-based). Two examples of data curation at TAIR are described below to illustrate our approach.

The first example, literature curation, illustrates an approach in which a software tool assists in manual curation of many small bits of data dispersed within the published literature. The goal is to annotate genes and gene products using controlled vocabularies based on information extracted from the literature [1]. To facilitate this process we have developed a literature curation software package, PubSearch (**pubsearch.org**), which stores genes, publications and controlled vocabulary terms, and provides a web user interface for manual curation. PubSearch's source code is available from Sourceforge (**www.gmod.org**).

Copyright © 2004 John Wiley & Sons, Ltd. 

The literature curation procedure starts with the collection of publications that contain the term '*Arabidopsis*' in the title or abstract. The titles and abstracts are then programmatically scanned to index publications with gene names and keywords. Because some matches to gene names may not be valid, curators subsequently validate the matches to genes manually. The validated associations of genes to papers are used to describe the gene function. One way of describing gene function is to assign controlled vocabulary terms that describe the gene product's molecular function, biological process and cellular location, as well as where and when the gene is expressed. Detailed description of the controlled vocabulary annotation is presented elsewhere [1].

The second example, curation of microarray data, illustrates an extensive manual curation and computational analysis of a single large dataset. This dataset was generated by the *Arabidopsis* Functional Genomics Consortium (AFGC) microarray project (**www.arabidopsis.org/info/2010_projects/comp_proj/AFGC/RevisedAFGC/site2L.htm**). Microarray data can be separated into two categories: metadata and numeric expression values. The metadata includes information about experimental design, RNA samples, array design, array elements and protocols. The array elements, array designs and slide descriptions were retrieved from the Stanford Microarray Database (**genome-www5.stanford.edu**). Additional information about RNA samples and experiments was collected from the AFGC website as text files. Metadata from both sources were organized and grouped into logical sets, and loaded into TAIR. Slides were grouped into replicate sets, and replicate sets were grouped into experiment sets. The experiment sets were annotated and categorized using controlled vocabularies. Annotation categories included experimental goal, experimental variable and experiment type. RNA samples were merged whenever possible to eliminate redundancy and were annotated with controlled vocabularies. Description of the growth conditions and treatments for each sample were standardized. Clone and sequence errors were identified and corrected and other missing data, such as sequence and species information, were added. Also, those array elements with available sequence information were mapped to the *Arabidopsis* genome by BLAST for each genome release.

The numeric expression values were subjected to the following quality control procedures: (a) slides with intensity values that were negative or zero at more than half of the spots after background subtraction were removed; and (b) if any spot was flagged as unreliable either by software or experimenter (e.g. bad PCR result), its data were excluded from further analysis. Data that passed the quality control filtering were normalized using a sub-array or the print-tip-group 'lowess' method [13]. This step practically eliminated strong spatial bias from 57 arrays, as determined by one-way analysis of variance (ANOVA) calculations. The resulting normalized, background-subtracted data were used to calculate logarithm (base 2) of the ratio of the mean intensity of Channel 2 to Channel 1 and fold change. We also determined standard error for the array elements that were spotted more than once on the array and calculated the average logarithm (base 2) of the ratio, fold change and standard error for each set of replicated slides.

### Results

### Size of database and usage statistics

Since its inception in September 1999, TAIR has grown dramatically in usage, impact and quantity and variety of data. In the past 4 years we provided approximately 300 Gb of information (18 million page views) to 834 000 unique IP addresses via the Web. Currently (March 2004), we provide about 900 000 page views to about 27 000 unique IP addresses per month, an approximately 10-fold increase in usage from the previous *Arabidopsis* database, AtDB [9]. Currently there are 12 752 registered users and 4745 laboratories, making our user group one of the largest organism-based biological research communities (Figure 3).

### Database content

Currently, TAIR stores 33 Gb of data (20 Gb in the database and 13 Gb on the FTP site), a size corresponding to about 28% of the latest version of GenBank database (version 138.0, 118 Gb). We have incorporated data from the *Arabidopsis* genome sequencing project and functional genomics projects, published literature, individual user submissions, and legacy data from AtDB. TAIR's major data types include clones

Copyright © 2004 John Wiley & Sons, Ltd. 

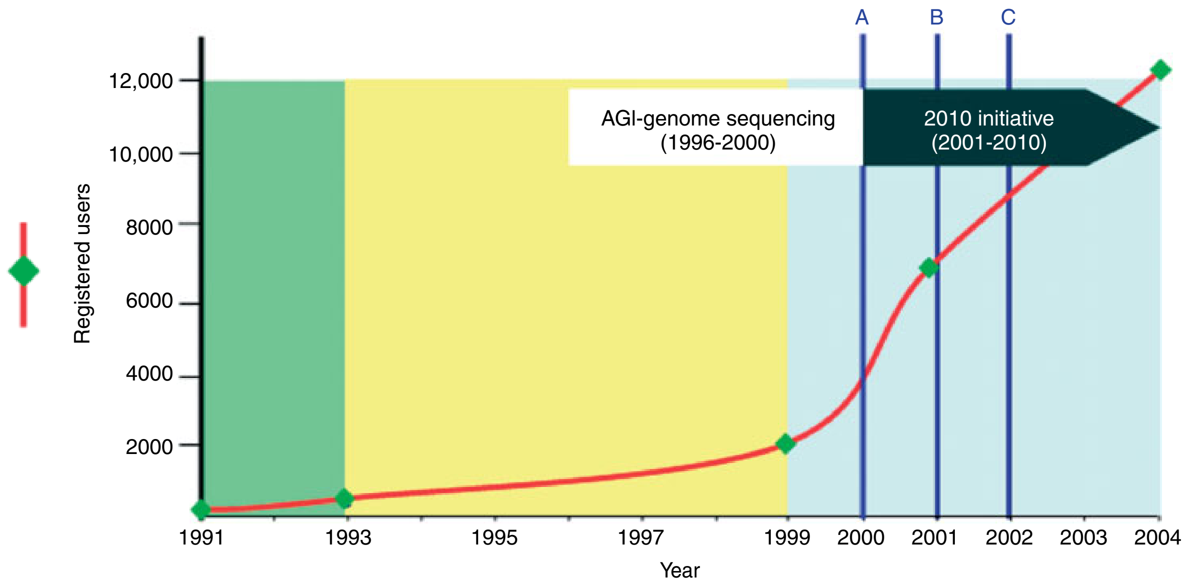

**Figure 3.** A short history of the *Arabidopsis* database. The *Arabidopsis* model organism database has undergone three transitions: 1991–1993 (green) AAtDB (an *Arabidopsis thaliana* Database) at Harvard/MIT; 1993–1999 (yellow) AtDB (*Arabidopsis thaliana* Database) at Stanford University; 1999–present (blue) TAIR (the *Arabidopsis* Information Resource) at Carnegie Institution, Department of Plant Biology and National Center for Genome Resources. The number of registered users is shown as a red line. Some landmarks are displayed as vertical blue lines. (A) Release of the *Arabidopsis* genome sequence by the *Arabidopsis* Genome Initiative (AGI) (B) TAIR merges with *Arabidopsis* Information Management System (AIMS), the database for *Arabidopsis* Biological Resource Center (ABRC) (C) TAIR hosts the first completed *Arabidopsis* functional genomics project, *Arabidopsis* Functional Genomics Consortium (AFGC)

(349 805 entries), clone ends (260 023 entries), genetic markers (3700), polymorphisms (258 847, including 205 650 tDNA insertions), germplasm accessions (198 019), stocks (383 520), publications (21 498), sequences (648 632), gene models (37 395), proteins (32 345), vectors (147), attributions (2 967 753), functional annotations (99 668), microarray data (515 slides) and microarray expression data points (over 6 million).

### Data curation

TAIR has made substantial progress in both manual and automated functional annotation of the *Arabidopsis* genome. We have been using controlled vocabularies (CV) such as Gene Ontology (GO) terms developed with the GO Consortium (**www.geneontology.org**) and TAIR-developed terms for *Arabidopsis* anatomy and developmental stages to describe the subcellular location of a gene product, its function, the biological processes it is involved in, and the anatomical parts and developmental stages in which the gene is expressed [1]. Each assignment of a CV term to a gene is associated with both an evidence code and an evidence description that detail the type of evidence used to make the assignment (ranging from strong experimental evidence, such as direct enzyme assay, to computational prediction). As of April 2004, we have assigned at least three controlled vocabulary terms to nearly all of the approximately 5870 *Arabidopsis* genes identified in the titles and abstracts of about 9105 papers obtained from PubMed, Agricola and BIOSIS. More than 500 genes have annotations describing their protein and/or mRNA expression patterns from the literature. In addition to our manual literature curation, we have used computational methods to assign CV terms to the whole genome complement, including many genes that have not been described in the literature.

### Software infrastructure

We invested a considerable effort into structuring the TAIR database and software for flexibility and accurate representation of complex data relationships. The TAIR system is

Copyright © 2004 John Wiley & Sons, Ltd. 

built on industry-standard relational database software (Sybase) and data access software written in Java, using Servlets and Java Server Pages (JSP) technologies. The database currently has about 200 tables in 16 knowledge domains, including general metadata, genome assignment, map element, community, reference, genes, germplasm, keywords, microarrays, polymorphisms, genetic markers, stocks and sequences (**arabidopsis.org/search/schemas.html**). For efficiency of software development and maintenance, the TAIR software is built in three logical tiers, consisting of a data layer, a business rules/logic layer and a user interface (UI) layer (Figure 2B).

### Tools

To provide our users with powerful ways to view the large data holdings within TAIR, we developed several query, browse and visualization software tools using Java Servlets. These include: (a) advanced search/browse pages (**arabidopsis.org/servlets/Search?type=general&action=new_search**); (b) SeqViewer (**arabidopsis.org/servlets/sv**), which allows the visualization of the sequenced genome and all objects mapped onto the genome from the chromosome level down to the nucleotide level; (c) MapViewer (***arabidopsis*.org/servlets/mapper**), which displays sequence-based, physical and genetic maps together; and (d) Keyword Browser (**arabidopsis.org/servlets/Search?action=new_search&type=keyword**), which allows the visualization of biological concepts along with annotations to data objects in a collapsible/expandable hierarchical tree format. In addition to developing new tools at TAIR, we took advantage of existing software for visualization of complex data, e.g. we used Pathway Tools software to provide detailed graphical views of metabolic pathways down to the molecular structure of metabolites in a reaction (**arabidopsis.org/tools/aracyc/**) [6]. Similarly, we filtered, normalized and clustered all of the microarray data in the TAIR database and made them available for mining using VxInsight, a 3-D terrain visualization software package (**arabidopsis.org/tools/bulk/microarray/analysis/index.jsp**) [2]. We have also developed software and analysis pipelines to systematically capture and annotate the genome with information from published articles and numerous genomic and functional genomics projects.

### Collaborations

During the first 4 years we established a large number of collaborations with researchers, other biological databases and commercial organizations. One of the most significant of these was the incorporation of database functions for the *Arabidopsis* Biological Resource Center (ABRC) [11] into TAIR. ABRC is an *Arabidopsis* stock center at Ohio State University that maintains and distributes seed and DNA stock to researchers. The collaboration between TAIR and ABRC allows users to use the query tools of TAIR to locate ABRC stock and purchase the stock immediately via the TAIR user interface. We also collaborated with Cereon Genomics, a subsidiary of Monsanto, to release over 80 Mb of *Landsberg erecta* sequence and over 57 000 SNPs to the public [5]. To our knowledge, this was the first successful collaboration between the private sector and a publicly funded resource to make privately funded data freely available to the public. We have been involved in extensive collaborations with other biological databases and established community standards in controlled vocabulary development and software development (e.g. Gene Ontology Consortium, **www.geneontology.org**; Plant Ontology Consortium, **www.plantontology.org**; and Generic Model Organism Database, **www.gmod.org**). Finally, we have collaborated extensively with TIGR in setting standards for data exchange of genome annotation information.

### Discussion

Recent technical advances in large-scale sequencing and genomics methods have triggered a scientific revolution with immense potential for extending biological knowledge. They have also posed an immense challenge: how to make optimal use of vast quantities of biological data. Without long-term high quality mechanisms for accessing and analysing the data, the resources used in generating the data are in danger of going to waste. In addition, in the absence of resources to make connections between previously existing and new data, the data already gathered will never be transmuted into knowledge but will remain as isolated sets of facts. It is essential that databases to house the data, tools and interfaces to provide access to the data,

Copyright © 2004 John Wiley & Sons, Ltd. 

and curatorial efforts to organize data for maximum utility, be established and maintained for the long term.

We designed and implemented a comprehensive information resource for *Arabidopsis* research. During this process, we learned a number of useful lessons. One of these was that planning time should not be underestimated. Thorough analysis of specifications and requirements, and research into available technologies, knowledge and expertise, were critical in the effective implementation of software. Also, explicit and clear communication among biologists and programmers was essential. We also learned that, while matrix organization (a person belonging to several projects and a project having several members) is unavoidable, minimization of the number of projects per person at a given time was helpful. In addition, dedicating resources for maintenance and enhancement is of great importance, since most of the data and usage are highly dynamic. Finally, we found that collaborating with other groups that are engaged in solving similar problems was extremely productive.

Biological databases such as TAIR can affect the informatics fields in four significant ways: (a) as a model system architecture and infrastructure for other databases; (b) as a source of standard operation procedures for systematic curation of experimental data; (c) as a driving force for establishing standard data exchange formats and mechanisms; and (d) as provider of current and complete datasets coupled with up-to-date experimentally verified information for computational analyses.

The capture, curation and presentation of a comprehensive set of information for *Arabidopsis* at one site relieve individual researchers of the need to engage in the same activities on their own. The time saved can be redirected to critical analyses of the information and to generating and testing hypotheses in the researchers' domain of expertise. In the year 2003, over 11% (143/1261) of *Arabidopsis* papers mentioned TAIR, making TAIR a project with a highly visible impact on *Arabidopsis* research.

### Acknowledgements

We are grateful to all of the members of the former and current TAIR team. The current members are Leonore Reiser, Tanya Berardini, Suparna Mundodi, Peifen Zhang, Nick Moseyko, Katica Ilic, Brandon Zoeckler, MG-H, EH, SYR (curators), Danny Yoo, Iris Xu, Mary Montoya, Jessie Zhang, Thomas Yan, DW, NM (programmers), Chris Somerville, Bill Beavis, Chris Town, David Meinke, Randy Scholl (executive committee). We are particularly grateful to our unsung heroes: Julie Tacklind (webmaster), Forrest Black, John Utsey, Glenn Ford, Faye Schilkey (systems and database administrators). This project was supported in part by NSF Grant No. DBI-9978564. This is Carnegie Publication No. 1675.

### References

1. Berardini T, *et al.* 2004. Functional annotation of the *Arabidopsis* genome using controlled vocabularies. *Plant Physiol* (in press).
2. Boyack KW, Wylie BN, Davidson GS. 2002. Domain visualization using VxInsight for science and technology management. *J Am Soc Inf Sci Technol* **53**: 764–774.
3. Gish W. 1996–2004. **http://blast.wustl.edu**.
4. Goff SA, Ricke D, Lan TH, *et al.* 2002. A draft sequence of the rice genome (*Oryza sativa* L. ssp. *japonica*). *Science* **296**: 92–100.
5. Jander G, Norris SR, Rounsley SD, *et al.* 2002. *Arabidopsis* map-based cloning in the post-genome era. *Plant Physiol* **129**: 440–450.
6. Karp PD, Paley S, Romero P. 2002. The Pathway Tools software. *Bioinformatics* **18**(suppl 1): S225–S232.
7. Meinke DW, Cherry JM, Dean C, Rounsley SD, Koornneef M. 1998. *Arabidopsis thaliana*: a model plant for genome analysis. *Science* **282**: 662, 679–682.
8. Palmer LE, Rabinowicz PD, O'Shaughnessy AL, *et al.* 2003. Maize genome sequencing by methylation filtration. *Science* **302**: 2115–2117.
9. Rhee SY, Weng S, Bongard-Pierce DK, *et al.* 1999. Unified display of *Arabidopsis thaliana* physical maps from AtDB, the *A.thaliana* database. *Nucleic Acids Res* **27**: 79–84.
10. Rhee SY, Beavis W, Berardini TZ, *et al.* 2003. The *Arabidopsis* Information Resource (TAIR): a model organism database providing a centralized, curated gateway to *Arabidopsis* biology, research materials and community. *Nucleic Acids Res* **31**: 224–228.
11. Scholl RL, May ST, Ware DH. 2000. Seed and molecular resources for *Arabidopsis*. *Plant Physiol* **124**: 1477–1480.
12. Somerville C, Dangl J. 2000. Genomics. Plant biology in 2010. *Science* **290**: 2077–2078.
13. Yang YH, Dudoit S, Luu P, *et al.* 2002. Normalization for cDNA microarray data: a robust composite method addressing single and multiple slide systematic variation. *Nucleic Acids Res* **30**: e15.
14. Young ND, Mudge J, Ellis TH. 2003. Legume genomes: more than peas in a pod. *Curr Opin Plant Biol* **6**: 199–204.
15. Yu J, Hu S, Wang J, *et al.* 2002. A draft sequence of the rice genome (*Oryza sativa* L. ssp. *indica*). *Science* **296**: 79–92.

Copyright © 2004 John Wiley & Sons, Ltd. 
